# A Comparison of Sprint Mechanical Parameters Measured With Timing Gates and a Laser Gun

**DOI:** 10.3389/fspor.2022.877482

**Published:** 2022-04-13

**Authors:** Roland van den Tillaar, Markus Estifanos Haugen, Hallvard Nygaard Falch

**Affiliations:** Department of Sports Science, Nord University, Levanger, Norway

**Keywords:** force-velocity profiling, team sports, Musclelab^TM^, F-V analysis, time correction

## Abstract

The aim of the study was to compare sprint mechanical parameters measured with timing gates and a laser gun. Thirty-four female team handball players (age: 17.0 ± 2.3 years, height: 1.70 ± 0.07 m, body mass: 66.7 ± 9.7 kg) performed three 30 m sprints in which the times were measured at 5, 10, 20 and 30 m with timing gates (accuracy 0.01 s) together with the distance over time by a laser gun. The main findings were that with a correction of +0.21 s (timing gates) the times and sprint mechanical properties calculated with the spreadsheet of Morin between timing gates and laser gun were not different. But when peak velocity was derived directly from the laser gun (Musclelab^TM^ system) this was significantly higher than maximal velocity (v_max_), and lower than the theoretical maximal velocity (v_0_) calculated with the spreadsheet. It was concluded that a correction of +0.21 s should be used to get correct mechanical properties when measuring with timing gates compared with laser gun measurements on an indoor court.

## Introduction

One of the key factors in many team sports is the individual athlete's capacity to sprint fast. To sprint at high velocity, the athlete should be able to accelerate quickly, as well as being able to reach a high peak velocity. This applies to both track and field events and team sports like soccer, team handball, and American football. This ability to sprint has been related to the athlete's capacity to apply large amounts of force in the horizontal direction (high propulsive power). To describe this mechanical capability, a simple field method has been developed to compute power, force, and velocity outputs of sprint performance in athletes (Morin and Samozino, 2016; Samozino et al., 2016). The method is based upon an inverse dynamic approach applied to the body's center of mass during maximal overground sprint acceleration, using only anthropometric (body mass and height) and spatiotemporal data (step time and length). The key mechanical parameters include the theoretical maximal horizontal force (F_0_), the theoretical maximal velocity (v_0_), and the ratio between these two parameters (Morin et al., 2011, 2012; Rabita et al., 2015). These outcome properties are then used to identify the weaknesses of the sprint performance, and address these weaknesses through training (Rakovic et al., 2018; Haugen et al., 2019).

In order to measure the horizontal force, force plates are considered to be the gold standard, but the use of a force plate is both expensive and requires a lot of logistics like embedded in the track for good measurements. Due to these challenges with force plates this method is not much used in testing in team sports, especially not in sports with limited finances (Samozino et al., 2016; Morin et al., 2019). A much cheaper method is the use of several timing gates at different distances, in which the split times are used to indirectly calculate these sprint mechanical parameters as shown by Samozino et al. (2016). However, when using timing gates, one major limitation is the lag in time between the first instance of force production and when the gates are triggered, which happens at the beginning of the sprint. This results in an overestimation of several parameters, such as force (F_0_) and velocity (v_0_) as shown by Vescovi and Jovanović (2021). In order to minimize this, several studies have recommended different time corrections. One of +0.5 s has been recommended (Haugen et al., 2019, 2020b), but also +0.3 (Vescovi and Jovanović, 2021), +0.27 (Haugen et al., 2012), and +0.25 s (Vescovi and Jovanović, 2021) were suggested as corrections. These differences in time correction were based on different start methods, such as from a standing start or a starting block (Haugen et al., 2012), and the distance from initiating the start of timing gate of 0.05 m (Vescovi and Jovanović, 2021) vs. 0.50 m (Haugen et al., 2019, 2020b). It was observed that as the distance between the start and initiation of the timing gate decreased the correction time decreased (0.5 vs. 0.25 s).

Yet, all of these studies did not compare their model directly with the gold standard of force plates, or radar and laser technology. A radar or laser measures the displacement over time, and thereby indirectly captures horizontal forces and the sprint mechanical properties as a field-based method (Buchheit et al., 2014; Marcote-Pequeño et al., 2019; Edwards et al., 2020). One such system is Musclelab^TM^, which is a system that uses a laser to measure the sprint distance over time, automatically calculating the different sprint mechanical properties, as well as the times at different sprint distances. As suggested by Vescovi and Jovanović (2021), it is important to compare the sprint mechanical properties measured by timing gates with a laser gun. As an overestimation of the sprint mechanical properties measured without correction with timing gates can result in describing wrong training programs (more training on peak velocity, while more strength is necessary when time-corrected) to the athletes, based upon these sprint mechanical properties. Furthermore, is it important to determine whether these properties automatically calculated with a laser system are comparable with the properties calculated with times at different distances by using the spreadsheet of Morin (Samozino et al., 2016; Morin et al., 2019). The spreadsheet of Morin makes it possible, by using split time measurements from an all-out sprint acceleration, to calculate automatically the sprint mechanical properties and validates against force plate data of Samozino et al. (2016).

Therefore, the aim of this study was twofold. First, it compared the sprint mechanical properties calculated with the spreadsheet of Morin (Samozino et al., 2016; Morin et al., 2019) between timing gates, the times over distance measured with a laser gun, and the corrected times of those measured between the two systems. Second, the aim was to compare the sprint mechanical properties automatically calculated with the Musclelab^TM^ system and those based on the times over different distances measured with the laser and calculated with the spreadsheet of Morin and Samozino (2019). It was hypothesized that with the time correction, the sprint mechanical properties between the two systems should not be different, and that the automatically calculated sprint mechanical properties measured with Musclelab^TM^ would be identical to those calculated with the spreadsheet, as they were based on the same numbers.

## Methods

### Participants

Thirty-four young female handball players (age: 17.0 ± 2.3 years, height: 1.70 ± 0.07 m, body mass: 66.7 ± 9.7 kg, training experience: 9±1.2 years) playing at the highest junior level of the country participated in the study. All the subjects were informed of the risks and benefits of participation, and a written consent form was obtained before the test from the participants and parents (when the participants were under 18). The study was conducted according to ethical regulations for research approved by the Norwegian Centre for Research (project approval: 903955) and in line with the latest revision of the Declaration of Helsinki.

### Procedure

All participants underwent a familiarization session in which the same procedure was used as during the test session. Before testing sprint performance, a standardized warm-up protocol of 10 min by van den Tillaar et al. (2019) was performed. This consisted of eight sub-maximal runs of 40 m with increasing intensity with dynamic stretching in between each run. After the warm-up, three attempts of 30 m maximal sprints were performed with 6–8 min rest between each attempt. Sprint times were measured with five pairs of wireless photocells (Brower Timing Systems, Draper, USA) at the start, 5, 10, 20, and 30 m distances with a 0.01 s accuracy. Participants initiated each sprint from a standing start in a split stance with the lead foot behind a line taped on the floor 0.05 m from the first pair of photocells which had a height of 1 m (to avoid that the upper body breaks the beam to early when taking start position), while the rest were at 1.2 m; this was similar to the study of Vescovi and Jovanović (2021). Velocity measurements were recorded continuously during each attempt using a laser gun (CMP3 Distance Sensor, Noptel Oy, Oulu, Finland), sampling at 2.56 KHz. A polynomial on distance over time was fitted, and automatically resampled over 1,000 Hz by Musclelab^TM^ v10.212.98 (Ergotest Technology AS, Langesund, Norway). The software automatically calculated peak velocity, the distance at which peak velocity was reached, peak force per body mass (N/kg), and force-velocity ratio. In addition, for each sprint it also automatically calculated the times at each five meters of the 30 m sprint test and used these measurements for further analysis. All sprints were conducted in an indoor hall on a surface of Polyurethane, and the participants used their regular indoor handball shoes.

To compare the sprint mechanical parameters: the theoretical maximal horizontal force (F_0_), the theoretical maximal velocity (v_0_) were calculated based upon the sprint times at 5, 10, 20, and 30 m measured with the timing gates and with the laser system by using the spreadsheet provided downloaded from JB Morin's homepage (https://jbmorin.net/2017/12/13/a-spreadsheet-for-sprint-acceleration-force-velocity-power-profiling/). Furthermore, F_0_ and v_0_ were also calculated when the 5 m times were corrected with the main difference over all three sprints from all subjects between the 5 m times measured with the laser gun compared with the timing gates.

### Statistical Analyses

Normality was tested using the Shapiro–Wilks test. To compare the sprint mechanical parameters between the two systems, a one-way analysis (timing gates, corrected timing gates, and laser gun) of variance (ANOVA) was used with repeated measures for each parameter. The level of significance was set at *p* < 0.05, and all data were expressed as mean ± standard deviation (SD). Analysis was performed with SPSS Statistics for Windows, version 25.0 (IBM Corp., Armonk, NY, USA). Effect size was evaluated with partial eta squared ($\eta_{\text{p}}^{2}$) where 0.01 < $\eta_{\text{p}}^{2}$ ≤ 0.06 constituted a small effect, 0.06 < $\eta_{\text{p}}^{2}$ < 0.14 a medium effect, and $\eta_{\text{p}}^{2}$ ≥ 0.14 a large effect (Cohen, 1988).

## Results

According to the Musclelab^TM^ system, peak velocity was on average 6.82 ± 0.37 m/s and reached at 22.57 ± 3.17 m from the start. The peak force per body mass was calculated as 6.34 ± 0.57 N/kg, resulting in a calculated force-velocity ratio of 0.93 ± 0.07 (Table 1).

**Table 1:** Mean (±SD) peak force and velocity and force-velocity ratio was measured and automatically calculated with the Musclelab^TM^ system, together with the distance at which maximal velocity was reached over all subjects.

	**Peak velocity**	**Peak force**	**Distance peak**	**Force-velocity**
	**(m/s)**	**(N/kg)**	**velocity (m)**	**ratio**
Laser gun	6.84 ± 0.37	6.35 ± 0.55	22.57 ± 3.17	0.92 ± 0.12

The difference in times at 5 m between the laser gun measurements and the timing gates was on average over all participants 0.21 ± 0.09 s. This was used as correction for the 5 m time and the following times in order to calculate the sprint mechanical properties (Table 2). A significant effect for the times and sprint mechanical properties was found between the uncorrected timing gates and the laser gun (F ≥ 70.6, *p* < 0.001, $\eta_{\text{p}}^{2}$ ≥ 0.45, Table 2). However, this was not observed between the corrected times and the laser gun (*p* ≥ 0.085, Table 2), when using the spreadsheet.

**Table 2:** Mean (±SD) sprint times and mechanical sprint properties measured with the timing gates, corrected, and the laser gun.

	Timing gates^*^	**Laser gun**	**Timing gates**
			**(corrected)**
5 m time (s)	1.33 ± 0.08	1.54 ± 0.11	1.54 ± 0.08
10 m time (s)	2.20 ± 0.11	2.41 ± 0.15	2.42 ± 0.11
20 m time (s)	3.73 ± 0.18	3.97 ± 0.21	3.94 ± 0.18
30 m time (s)	5.22 ± 0.26	5.42 ± 0.26	5.43 ± 0.26
F_0_ (N/kg)	9.51 ± 1.21	6.49 ± 0.85	6.50 ± 0.66
v_0_ (m/s)	6.81 ± 0.42	7.07 ± 0.42	7.10 ± 0.42
v_max_ (m/s)	6.62 ± 0.36	6.78 ± 0.37	6.82 ± 0.39
Force-velocity ratio	1.39 ± 0.20	0.91 ± 0.14	0.91 ± 0.11

* *indicates a significant difference with the laser gun on all parameters on a p < 0.05 level*.

When comparing the sprint mechanical properties automatically calculated with Musclelab^TM^, with the parameters calculated by the spreadsheet of Morin https://jbmorin.net/2017/12/13/a-spreadsheet-for-sprint-acceleration-force-velocity-power-profiling/) measured by laser or corrected times from timing gates, F_0_ (*F* = 2.0, *p* = 0.134, $\eta_{p}^{2}$ = 0.03) and force-velocity ratio (*F* = 01, *p* = 0.9, $\eta_{p}^{2}$ = 0.01) were not significantly different. However, the peak velocity automatically calculated with the Musclelab^TM^ system was significantly higher (Table 1) than the v_max_ and lower than the v_0_ calculated with the spreadsheet (Table 2), based on the corrected times and the laser gun times (F ≥ 14.3, *p* < 0.01, $\eta_{\text{p}}^{2}$ ≥ 0.15).

## Discussion

The purpose of the present study was to compare the sprint mechanical properties calculated with the spreadsheet of Morin (Samozino et al., 2016; Morin et al., 2019) between timing gates with the times over distance measured with a laser gun, and the corrected times between those measured between the two systems and the sprint mechanical properties automatically calculated with the Musclelab^TM^ system. The main findings were that with a correction of +0.21 s the times and sprint mechanical properties calculated with the spreadsheet of Morin between the systems were similar. But peak velocity from the automatically calculated Musclelab^TM^ system was significantly higher than the v_max_ and lower than the v_0_ calculated with the spreadsheet of Morin et al. (2019).

In the present study the correction was +0.21 s, which was lower than in the previous studies, corrections varying from +0.25 to +0.5 s (Haugen et al., 2020a,b; Vescovi and Jovanović, 2021) as timing gate correction. The lower correction was due to the distance from initiation to the starting gate of 0.5 m (Haugen et al., 2020a,b) vs. only 0.05 m, and the running surface indoor court compared to sprinting on turf (Vescovi and Jovanović, 2021). Sprinting on an indoor handball court causes more friction than sprinting on a turf surface, and therefore the difference between initiation could be increased by +0.04 s. Then again, the correction of +0.21 s resulted in the same times at the different distances and the sprint mechanical properties as measured with the laser gun. Therefore, it is suggested that this correction should be used when testing female handball players on an indoor court.

The sprint times in the present study were slower than those of a similar study with timing gates (Vescovi and Jovanović, 2021). This was due to a difference in the age and experience of the subjects. In the present study the main age was 17 vs. 23 years. Furthermore, the subjects in the present study were female handball players, who mainly sprint only 10–20 m (Michalsik and Aagaard, 2015) due to the total length of the court, while the subjects in the study of Vescovi and Jovanović (2021) were soccer players that sprint longer distances during a match and training thereby they probably learn to accelerate longer (Vescovi and Favero, 2014). Furthermore, due to the differences in sprinting times, the sprint mechanical properties in the present study (F_0_: 6.5 ± 0.85 N/kg and v_0_: 7.07 ± 0.42 m/s) were lower than in previous studies (F_0_: 7.1–7.6 N/kg and v_0_: 8.0–8.1 m/s) (Haugen et al., 2020b; Vescovi and Jovanović, 2021).

Comparing the sprint mechanical properties automatically calculated by the Musclelab^TM^ with the ones calculated with the spreadsheet of Morin et al. (2019), resulted in similar F_0_ and force-velocity ratio numbers which were therefore directly comparable. However, the peak velocities calculated with the spreadsheet of Morin were on average 0.06 and 0.025 m/s (Table 2) lower than those measured with the Musclelab^TM^ system, even when the times over the different distances were calculated directly from the laser system. These lower peak velocities from the spreadsheet are explainable by the fact that the polynomial velocity–time curve in the spreadsheet was based on four data points (timed at 5, 10, 20, and 30 m) and the peak velocity was estimated on these four data points. The laser gun provided velocity measurements with a higher sampling rate, which results in more accurate reading. Furthermore, the peak velocity was not estimated, but observed. Therefore, a correction of adding 0.02 to 0.06 m/s should be made for the peak velocity when comparing sprint mechanical properties calculated with the spreadsheet of Morin and the sprint mechanical property results generated automatically by Musclelab^TM^.

These sprint mechanical properties were based upon 30 m sprints, which could be a limitation of the study as it is possible that subjects are not finished with their acceleration, and therefore, this underestimates their peak velocity and v_0_, as elite sprinters stop accelerating after 30–50 m (van den Tillaar, 2021). However, in the present study, the peak velocity was reached on average at 22.6 ± 3.17 m from the start. Thereby, no underestimation of v_0_ and v_max_ was to be expected. However, when testing male handball players, higher level players, and athletes that normally sprint over longer distances (e.g. in soccer or American Football) a longer testing distance is required. Thus, other time corrections could be necessary, and these should be applied in future studies. Also, the effect of the surface should be investigated, to study if sprinting on a different surface requires a different time correction to calculate the sprint mechanical properties, in order to be able to compare them with laser and radar systems, and to avoid overestimation.

## Conclusion

Based upon the findings of the present study, a correction of +0.21 s should be used to get correct mechanical properties when measuring female handball players with timing gates compared with laser gun measurements on an indoor court. Furthermore, peak velocity calculations based on the spreadsheet of Morin are always slightly lower than the peak velocity measured with a laser gun, due to the low number of data points the velocity–time curve is based on when using the spreadsheet. For coaches and athletes, this information on the mechanical properties measured with different systems is important when comparing results between measuring equipment, and for identifying potential weaknesses in sprinting ability.

## Data Availability Statement

The raw data supporting the conclusions of this article will be made available by the authors, without undue reservation.

## Ethics Statement

The studies involving human participants were reviewed and approved by Norwegian Centre for Research (project approval: 903955). Written informed consent to participate in this study was provided by the participants' legal guardian/next of kin.

## Author Contributions

Conception was done by RT. Data collection, acquisition, and writing was performed by MH, HF, and RT. All authors contributed to the article and approved the submitted version.

## Conflict of Interest

The authors declare that the research was conducted in the absence of any commercial or financial relationships that could be construed as a potential conflict of interest.

## Publisher's Note

All claims expressed in this article are solely those of the authors and do not necessarily represent those of their affiliated organizations, or those of the publisher, the editors and the reviewers. Any product that may be evaluated in this article, or claim that may be made by its manufacturer, is not guaranteed or endorsed by the publisher.
